# Focal HIFU therapy for anterior compared to posterior prostate cancer lesions

**DOI:** 10.1007/s00345-020-03297-7

**Published:** 2020-07-07

**Authors:** Philipp M. Huber, Naveed Afzal, Manit Arya, Silvan Boxler, Tim Dudderidge, Mark Emberton, Stephanie Guillaumier, Richard G. Hindley, Feargus Hosking-Jervis, Lucas Leemann, Henry Lewi, Neil McCartan, Caroline M. Moore, Raj Nigam, Chris Odgen, Raj Persad, Jaspal Virdi, Mathias Winkler, Hashim U. Ahmed

**Affiliations:** 1 Department of Urology, University Hospital Inselspital Berne, Bern, Switzerland; 2 Urologie St. Anna, Lucerne, Switzerland; 3 Department of Political Science, University of Zurich, Zurich, Switzerland; 4 Division of Surgery and Interventional Sciences, University College London, London, UK; 5 Department of Urology, UCLH NHS Foundation Trust, London, UK; 6 Imperial Prostate, Division of Surgery, Department of Surgery and Cancer, Faculty of Medicine, Imperial College London, Fulham Palace Road, London, W6 8RF UK; 7 Imperial Urology, Charing Cross Hospital, Imperial College Healthcare NHS Trust, London, UK; 8 Department of Urology, The Princess Alexandra Hospital NHS Trust, Harlow, UK; 9 Department of Urology, Dorset County Hospital NHS Trust, Dorchester, Dorset UK; 10 Department of Urology, University Hospital Southampton NHS Trust, Southampton, UK; 11 Department of Urology, Basingstoke Hospital, Hampshire Hospitals NHS Foundation Trust, Basingstoke, UK; 12 Springfield Hospital, Chelmsford, Essex UK; 13 Department of Urology, Royal County Surrey Hospital NHS Trust, Guildford, Surrey UK; 14 Department of Academic Urology, The Royal Marsden Hospital NHS Foundation Trust, London, UK; 15 Department of Urology, Southmead Hospital, North Bristol NHS Trust, Bristol, UK

**Keywords:** Prostate cancer, Focal therapy, High intensity focused ultrasound, Biochemical failure

## Abstract

**Objective:**

To compare cancer control in anterior compared to posterior prostate cancer lesions treated with a focal HIFU therapy approach.

**Materials and methods:**

In a prospectively maintained national database, 598 patients underwent focal HIFU (Sonablate^®^500) (March/2007–November/2016). Follow-up occurred with 3-monthly clinic visits and PSA testing in the first year with PSA, every 6–12 months with mpMRI with biopsy for MRI-suspicion of recurrence. Treatment failure was any secondary treatment (ADT/chemotherapy, cryotherapy, EBRT, RRP, or re-HIFU), tumour recurrence with Gleason ≥ 3 + 4 on prostate biopsy without further treatment or metastases/prostate cancer-related mortality. Cases with anterior cancer were compared to those with posterior disease.

**Results:**

267 patients were analysed following eligibility criteria. 45 had an anterior focal-HIFU and 222 had a posterior focal-HIFU. Median age was 64 years and 66 years, respectively, with similar PSA level of 7.5 ng/ml and 6.92 ng/ml. 84% and 82%, respectively, had Gleason 3 + 4, 16% in both groups had Gleason 4 + 3, 0% and 2% had Gleason 4 + 4. Prostate volume was similar (33 ml vs. 36 ml, *p* = 0.315); median number of positive cores in biopsies was different in anterior and posterior tumours (7 vs. 5, *p* = 0.009), while medium cancer core length, and maximal cancer percentage of core were comparable. 17/45 (37.8%) anterior focal-HIFU patients compared to 45/222 (20.3%) posterior focal-HIFU patients required further treatment (*p* = 0.019).

**Conclusion:**

Treating anterior prostate cancer lesions with focal HIFU may be less effective compared to posterior tumours.

## Introduction

Since high-intensity focused ultrasound (HIFU) was first started for the treatment of prostate cancer [1], treatment parameters have been refined from ablating the whole prostate gland to hemi-gland ablation, quadrant ablation and so-called ultra-focal ablation of cancerous areas [2]. Less than whole-gland approaches seem to confer reassuring medium-term cancer control with reduced side-effects and complications [3], provided patients are staged and selected appropriately [4].

The energy source for HIFU is usually placed in the patient’s rectum, with ultrasound waves having to traverse a number of tissue planes. There can be loss of energy by passing multiple tissue planes and large volumes of tissue between source and prostate tumour. There has been some concern as to whether HIFU is able to confer similar cancer control rates for treating anterior prostate tumours. Indeed, the reported local intraprostatic swelling and prostate shift during the treatment [5] may have a higher impact on anterior parts of the prostate, with the anterior border of the lesion drifting away from the energy. We aimed to compare cancer control in anterior compared to posterior prostate cancer lesions treated with a focal HIFU therapy approach.

## Methods

The UCLH Joint Research Office granted institutional review board exemption. Between March/2007 and November/2016, 598 consecutive patients underwent primary focal HIFU (focal lesion ablation or quadrant or hemiablation) for non-metastatic prostate cancer using the Sonablate®500 device (Sonacare Inc., USA) within nine centres. Hemiablations were excluded from this analysis. Prior to consensus articles which streamlined eligibility criteria for focal therapy in prostate cancer, it was not known nor agreed which cases may benefit most. As a result, focal HIFU treatment was initially offered to patients diagnosed with non-metastatic prostate cancer with Gleason 6 through 9, stage T1c-T3bN0M0 and PSA of ≤ 20 ng/ml. Gleason 6 required a minimum of 3 mm of disease. Over the last 5 years, the UK Focal Therapy Users Group predominantly treats men with stage T1c-T2cN0M0, Gleason 7 and PSA ≤ 20 ng/ml. For this analysis, only patients with Gleason ≥ 7 were included since positive recurrence was defined as Gleason ≥ 7 as described below. Furthermore, for all cases analysed prostate size in the group with tumour located in posterior area of the gland was limited to the maximum of prostate size of the comparator group. 267 patients were eligible for analysis in this study as they had more than 3 months follow-up and information on tumour location. Disease was localised using mpMRI, combined with targeted and systematic biopsies, or transperineal mapping biopsies. Cases were separated into anterior or posterior groups according to tumour location in mpMRI where each prostate gland was arithmetically divided in half in anterior–posterior dimensions.

Treatments included in this analysis were delivered in a focal lesion ablation or quadrant fashion depending on the gland volume as well as tumour volume and location. Index lesion ablation alone was conducted in patients with multifocal disease provided untreated areas harboured no more than 3 mm of Gleason 6 on systematic or template mapping biopsies. All men were advised to undergo 3–6 monthly serum PSA testing. An mpMRI was routinely performed regardless of PSA changes at 1 year and approximately 1–2 yearly thereafter. Two rises in PSA after the nadir level was achieved, without predefining the level of rise, was investigated with a prostate biopsy, or mpMRI followed by biopsy if the mpMRI was suspicious. We have previously reported on the high negative predictive value of mpMRI in the post-focal HIFU setting for clinically significant prostate cancer [6]. Clinically significant cancer on biopsy of untreated areas was defined as ‘out-of-field’ progression.

Treatment failure was defined by any secondary treatment (ADT/chemotherapy, cryotherapy, EBRT, RRP, or re-HIFU), metastasis from prostate cancer without further treatment, tumour recurrence with Gleason score ≥ 7 proved by prostate biopsy without further treatment, or death from prostate cancer, respectively.

### Statistical analysis

Variables with skewed distribution are reported as median (interquartile ranges, IQR). Categorical variables are reported as absolute numbers with percentages. Significance levels for median values were calculated with Mood’s Median Test, and treatment results with Fisher’s Exact Binomial Test. Multivariate analysis was performed with logit models using the following factors: age, PSA level pre-treatment, and prostate volume were used as continuous parameters, whereas tumour location (anterior vs. posterior), clinical T stage (< cT3 vs. ≥ cT3), and Gleason score (< 4 + 3 vs. ≥ 4 + 3) were categorised. Analyses were performed using the R language environment for statistical computing. *p* < 0.05 was considered as statistically significant.

## Results

### Baseline demographics

From a total of 598 focal primary HIFU cases, and applying eligibility criteria 267 patients were analysed, divided into a group of 45 cases with an anterior, and a group of 222 cases with a posterior located prostate tumour treatment (Table 1). Since prostate volume in posterior tumour group was limited to the maximum of prostate volume in anterior tumour group there was no significant difference. In the anterior group, no Gleason 4 + 4 was treated while in the posterior group 2% of cases were Gleason 4 + 4. The number of positive cores in biopsies pre-treatment was significantly higher in anterior tumour group compared to posterior tumour location (median 7 vs. 5, *p* = 0.009).

**Table 1:** Baseline demographics

Determinant	Anterior tumour		Posterior tumour		*p* value
	Median/mean/*N*	IQR/%	Median/mean/*N*	IQR/%	
Age at treatment (in years), median, (IQR)	64	59–68	66	60–71	0.531
ADT pre treatment, *N*, (%)	5	11%	24	11%	0.970
PSA pre treatment (in ng/ml), median, (IQR)	7.53	5.22–10.77	6.92	5.05–9.64	0.496
Prostate volume (MRI, in ml), mean, (range)	33	28–40	36	28–47	0.315
Biopsy results pre treatment, median, (IQR)					
*No. positive cores*	7	3–13	5	4–9	0.009
*MCCL (in mm)*	6.5	5–8.75	6	4–8	0.597
*Max. cancer percentage of core (%)*	50	40–76	60	39–75	0.507
Gleason score pre treatment, median	3 + 4		3 + 4		
*3* + *4*	38	84%	181	82%	0.832
*4* + *3*	7	16%	36	16%	1.000
*4* + *4*	0	0%	5	2%	0.593
T-stage pre treatment, median, *N*, (%)	T2		T2		
*T1c*	5	11%	10	4%	0.145
*T2a*	3	7%	15	7%	1.000
*T2b*	27	60%	117	53%	0.415
*T2c*	6	13%	50	22%	0.228
*T3a*	4	9%	28	13%	0.619
*T3b*	0	0%	2	1%	1.000

*N* number, *IQR* inter quartile range, *ADT* androgen deprivation therapy, *MRI* magnetic resonance imaging, *ml* millilitres, *MCCL* maximum cancer core length, *cm* centimetre

### Treatment outcome

The two groups showed statistically significant different failure rates. In anteriorly located tumours failure occurred in 17/45 cases (37.8%), whereas in posteriorly located tumours 45/222 cases (20.3%) had a treatment failure (*p* = 0.019). There were no differences in time to treatment failure or follow-up without failure (Table 2). In multivariate analysis using logit models anterior tumour location and ≥ cT3 stage resulted as a significant predictor for treatment outcome (Table 3).

**Table 2:** Treatment outcomes

Determinant	Anterior tumour		Posterior tumour		*p* value
	Median/mean/*N*	IQR/range/%	Median/mean/*N*	IQR/range/%	
Total patients, *N*	45		222		
Recurrences in treated area, *N*, (%)	17	37.8%	45	20.3%	0.019
Proof of recurrence by					
*Biopsy, N, (% of recurrences)*	8	47%	22	49%	0.930
*mpMRI, N, (% of recurrences)*	9	53%	23	51%	1.074
Time to recurrence (mth), mean, (range)	28.5	22–41	21	18–36	0.274
Follow-up without recurrence (mth), mean, (range)	29	15.5–46	19	11–41	0.030
Secondary treatment modality					
*Secondary HIFU, N, (% of recurrences)*	9	53%	39	87%	
*Cryoablation, N, (% of recurrences)*	6	35%	0	0%	
*Radical prostatectomy, N, (% of recurrences)*	1	6%	5	11%	
*Radiotherapy, N, (% of recurrences)*	1	6%	1	2%	

*N* number, *IQR* inter quartile range, *mpMRI* multiparametric MRI, *mth* month, *ADT* androgen deprivation therapy

**Table 3:** Multivariate analysis: predictors for treatment failure

Determinant	OR	IQR	*p* value
Age at treatment	0.973	0.93–1.01	0.192
PSA pre-treatment	1.054	0.98–1.13	0.152
Prostate volume	0.992	0.97–1.02	0.526
Anterior tumour location	0.304	0.14–0.64	0.002
Clinical T stage ≥ cT3	3.831	2.03–7.39	< 0.001
Gleason score ≥ 4 + 3	1.754	0.80–3.75	0.152

*OR* odds ratio, *IQR* inter quartile range

## Discussion

In summary, we have shown that failure following focal HIFU is related to the position of the cancer in the anterior–posterior plane. Prior to discussing the clinical implications of our study, there are some limitations that require mentioning. First, it is a retrospective analysis of a national database with heterogeneity in case-mix. Second, there was not an underlying protocol, so we could not mandate biopsy in all men, particularly if the PSA was stable. Third, we lack long term evaluation of metastases and mortality and therefore used a composite endpoint [7]. Last, we were not able to evaluate whether apical versus mid-gland or base locations had impact on the failure rates.

Although focal treatment modalities have demonstrated their ability in treating prostate cancer [8], there are some technical limitations in HIFU. The probe used in our study (Sonablate500) has two pre-defined focal energy points at 4 cm and 3 cm lengths, there is an inherent limitation for glands which are large or have an AP length of much greater than 3.5 cm [9, 10]. What we have shown is that even when the probe can reach the lesion there is a relevant difference in successful treating of cancers localised more anteriorly compared to those closer to the rectal probe. There still seems to be an issue with ablative efficacy likely related to loss of energy as the pulse traverses multiple planes of tissue. With the high resolution and precision of a mpMRI for outlining prostate cancer boarders an accurate planning instrument is available [11]. The distance between rectal wall and the most anterior tumour border can be defined precisely, and unsuitable cases for HIFU with a tumour location beyond the maximal focal point have to be excluded. In our practice, we now use interstitial needle based ablative therapies [12, 13].

When planning a HIFU treatment for anterior prostate tumours one should bear in mind the intraprostatic changes, which are produced by heating energy through ablation. There is an intraprostatic swelling phenomenon that occurs as well as shifting of the prostate, demonstrated for whole-gland HIFU treatments [5], and even when a smaller area is treated focally, a certain shift of the targeted area away from the focal point has to be kept in mind.

Unlike in other focal ablative technologies—like cryotherapy, irreversible electroporation or laser approaches—where ablative energy can be delivered directly into a desired intraprostatic area, the HIFU energy source distributes its energy from an extra-prostatic point-source. Therefore, there is a higher loss of ablative energy in anterior located tumours, when more tissue needs to be crossed to reach the region of interest. For anterior areas, there is always more energy absorbed by posterior prostatic parenchyma than for posterior areas [14]. Whilst overall rates of cancer control are acceptable in the medium term in men with clinically significant prostate cancer [15], in this retrospective analysis a significantly higher recurrence rate for anterior located prostate cancers compared to posterior tumours is demonstrated. When comparing the underlying baseline demographics, there is a significant higher number of positive cores in the anterior tumour group. This difference must be a result of different number of total biopsy cores taken, since there was no protocol used how many cores have to be taken from suspicious lesions.

## Conclusion

Treating anterior prostate cancer lesions with focal HIFU may be less effective compared to posterior tumours.
